# Serum Lipopolysaccharide Is Associated with the Recurrence of Atrial Fibrillation after Radiofrequency Ablation by Increasing Systemic Inflammation and Atrial Fibrosis

**DOI:** 10.1155/2022/2405972

**Published:** 2022-10-15

**Authors:** Meng Wang, Hua Xiong, Li Lu, Tongjian Zhu, Hong Jiang

**Affiliations:** ^1^Department of Cardiology, Renmin Hospital of Wuhan University, Wuhan, China; ^2^Department of Cardiology, Xiangyang Central Hospital, Affiliated Hospital of Hubei University of Arts and Science, Xiangyang, China

## Abstract

**Objectives:**

The gut microbiota and its metabolites are linked to inflammation and contribute to the progression of atrial fibrillation (AF), but the predictive value of the gut microbiota-derived metabolite lipopolysaccharide (LPS) for AF recurrence (RAF) is unknown. This study is aimed at investigating (1) the correlation between LPS and RAF and (2) its relationship with inflammation and atrial fibrosis.

**Method:**

We performed a single-centre retrospective analysis in 159 AF patients. Fasting plasma samples were collected, and an enzyme-linked immunosorbent assay was used to determine the levels of serum LPS, interleukin-6 (IL-6), collagen type-1 C-terminal telopeptide (CITP), and transforming growth factor-*β*1 (TGF*β*1). The cumulative risk for RAF was evaluated with Kaplan–Meier analysis. Cox proportional hazard analysis was carried out to predict the hazard of RAF. The correlations among LPS and IL-6, CITP, TGF*β*1, and left atrial diameter (LAD) were analysed by Pearson's correlation coefficient. Subsequent univariate and multivariable linear regression analyses were carried out to evaluate the connection between clinical variables and Log-LPS.

**Results:**

All 159 AF patients were included in this study. The proportion of persistent atrial fibrillation was 40.3%, the mean age was 61.9 ± 10.1 years, the proportion of males was 61.6%, and the mean LPS was 56.5 ± 29.5 pg/mL. After all patients were divided into tertiles according to the circulating LPS level, a total of 44 RAF occurred: 10 in the first tertile, 15 in the second tertile, and 19 in the third tertile (log-rank test *P* = 0.037). Heart failure (hazard ratio 2.029, *P* = 0.041), LAD (hazard ratio 1.064, *P* = 0.022), Log-LPS (hazard ratio 5.686, *P* = 0.043), and CITP (hazard ratio 6.841, *P* = 0.033) independently predicted the risk of RAF. In all patients, univariate analysis showed that heart failure, LAD, hs-CRP, IL-6, CITP, and TGF-*β*1 were connected with Log-LPS. Multivariate linear regression analysis indicated that IL-6 and hs-CRP were independently and positively connected with Log-LPS.

**Conclusions:**

Our results indicated that circulating LPS was a predictor of RAF and may contribute to RAF incidence after ablation by increasing systemic inflammation and atrial fibrosis.

## 1. Introduction

Atrial fibrillation (AF) is associated with a high prevalence of arrhythmia, which is connected with heart failure, stroke, and mortality [1, 2]. Previous studies have shown that the gut microbiota and its metabolites are linked to inflammation and contribute to the progression of cardiovascular diseases [3]. Recent data have shown that gut microbiota-derived metabolites such as TMAO are connected with the occurrence and recurrence of atrial fibrillation (AF) [4]. Additionally, animal experiments have also shown that TMAO can promote the progression of AF by affecting systemic inflammation [5]. In addition, Li et al. [6] also demonstrated that a GM-based taxonomic scoring system can effectively predict the accuracy of AF recurrence (RAF) after primary ablation. Therefore, research is necessary to explore the effects of key gut microbiota-derived metabolites on RAF.

Lipopolysaccharide (LPS), a cell wall component from Gram-negative bacteria, is involved in various cardiovascular diseases [7]. Animal experiments have indicated that LPS can increase the expression levels of inflammatory cytokines and L-type calcium channel proteins as well as shorten the atrial effective refractory period (ERP), thereby promoting the occurrence of atrial fibrillation [8, 9]. Additionally, Zhang et al. [10] demonstrated that age-related gut dysbiosis induces AF by increasing serum LPS and glucose, which could activate the atrial NLRP3 inflammasome and promote atrial fibrosis. In addition, Pastori et al. found that circulating LPS is significantly associated with major adverse cardiovascular events (MACEs) in AF patients by increasing platelet activation [11]. However, the association between LPS and RAF has not been revealed. In this study, we aimed to assess the effect of circulating LPS on RAF during follow-up.

## 2. Materials and Methods

### 2.1. Study Population

All 159 enrolled patients were diagnosed with AF according to the guidelines of the European Society of Cardiology [12]. All patients were scheduled for radiofrequency catheter ablation procedures at Renmin Hospital of Wuhan University between February 2019 and February 2021. Major exclusion criteria were as follows: heart failure, any structural heart disease, stroke, infectious diseases, postsurgery status, acute coronary syndrome, renal or hepatic impairment, lipid lowering medication, any autoimmune disease, and other inflammatory diseases.

### 2.2. Serum Lipopolysaccharide

Fasting venous blood was centrifuged and stored at -80°C until biochemical determination. Serum LPS was determined with commercially available ELISA (Cusabio, Wuhan, China) following the instructions. The detection range of the kit was 6.25 pg/ml-400 pg/ml. The intra-assay and interassay precision were < 8% and < 10%, respectively. The absorbance of the sample was detected at 450 nm within 5 minutes. All serum samples were analysed in duplicate.

### 2.3. Inflammatory and Fibrotic Biomarkers

Serum samples were diluted twice, and the level of transforming growth factor beta1 (TGF-*β*1) was measured with commercially available ELISA (Cusabio, Wuhan, China). The value was read at 450 nm, and the detection range was 0.78 ng/mL-50 ng/mL. The interleukin-6 (IL-6) and collagen type-1 C-terminal telopeptide (CITP) levels were measured with a sandwich enzyme immunoassay with a commercially available ELISA (ELK Biotechnology), following the instructions. The intra- and interassay precision of all assays were < 8% and < 10%, respectively.

### 2.4. Laboratory Analysis

Fasting venous blood was obtained at baseline, and the levels of blood glucose (mmol/L), LDL (mmol/L), HDL (mmol/L), TC (mmol/L), TG (mmol/L), high-sensitivity C-reactive protein (hs-CRP, mg/L), and creatinine (*μ*mol/L) were determined through Dimension EXL with an LM automatic biochemical analyser (Siemens Healthcare Diagnostics).

### 2.5. Echocardiography

After admission, 2-dimensional transthoracic colour Doppler echocardiography was performed in all patients. Each echocardiographic result was analysed by two blinded experts. The left atrial diameter (LAD) was measured along the parasternal long-axis. The left ventricular (LV) diameters and wall thickness were measured, and the LV ejection fraction was quantitatively analysed according to the modified Simpson method.

### 2.6. Radiofrequency Catheter Ablation

The intracardiac electrograms were recorded by a computer-based electrophysiology system (Lead XP, Jinjiang Inc., Chengdu, China). We imported and constructed CT image of left atrium and pulmonary veins using CARTO Segmentation software (Biosense Webster, Inc.). Transseptal puncture was performed, and unfractionated heparin was started at 100 U/kg and thereafter at 1,000 U/h by intermittent boluses to maintain an activated clotting time > 250 s. The geometries of PVs were generated under the guidance of the Electroanatomical Mapping System (CARTO, Biosense Webster, Diamond Bar, CA, USA). Then, we merged the CT image and PV electroanatomical map to construct the left atrium anatomical model. Circumferential pulmonary vein isolation (CPVI) was performed using an irrigated catheter. At the endpoint of the procedure, the pulmonary vein spike potential and bidirectional block of the lines were assessed. Electrical cardioversion was performed in patients without restoring sinus rhythm.

### 2.7. Follow-Up

All participants were followed up for one year after ablation. In the first 3 months, amiodarone therapy was administered to prevent early atrial fibrillation recurrence. Patients with persistent AF receive oral anticoagulation (warfarin or new oral anticoagulants) for 3 months. After ablation, medical examination (24-hour Holter monitoring and 12-lead ECG) was routinely performed at 3, 6, 9, and 12 months. Additional ECGs are obtained if the patients have any suspected symptoms of RAF, such as palpitations and shortness of breath. RAF was defined as any atrial tachycardia lasting at least 30 s according to the ECG recording after the initial 3-month blanking period.

### 2.8. Statistical Analysis

All numerical analyses were conducted using SPSS 22.0 (SPSS, Inc., Chicago, IL, USA) and GraphPad prim 7.0 Software. The continuous variables are presented as the mean ± SD, and the differences were determined by Student's *t* test when these variables satisfied a normal distribution. If these variables determined by the Mann–Whitney U test were nonnormal distributions, these variables were presented as medians (interquartile range). The *χ*2 test or Fisher's exact test was carried out to compare the differences. All patients were divided into tertiles according to LPS level, and the Kaplan–Meier method was used to calculate the cumulative risk for RAF. The hazard of RAF was analysed using Cox proportional hazards analysis. Correlations among LPS and biomarkers of inflammation and atrial fibrosis were analysed by Pearson's correlation coefficient. Univariate and multivariable linear regression analyses were conducted to detect the clinical variables that were correlated with circulating LPS. Statistical significance was set as a *P* value < 0.05.

## 3. Results

### 3.1. Clinical Characteristics of AF Patients

The characteristics of all 159 enrolled AF patients are presented in Table 1. The mean age was 61.9 ± 10.1 years, 61.6% of the patients were male, and the proportion of persistent atrial fibrillation was 40.3%. The most common comorbidities were hypertension (53.5%), coronary heart disease (22.0%), and heart failure (21.4%). Compared with sinus rhythm (SR) maintenance patients, patients with AF recurrence had a greater LAD and higher levels of inflammatory and fibrotic biomarkers (Table 1).

### 3.2. LPS and RAF

**Figure 1:**
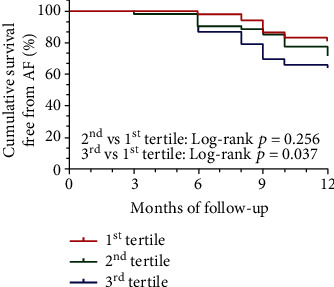
Kaplan–Meier curve for survival free from AF after ablation according to circulating LPS tertile.

During the 12-month follow-up period, 72.3% (115/159) of patients successfully maintained sinus rhythm. Of the 159 patients, the mean LPS was 56.5 ± 29.5 pg/mL, 25.2 ± 12.4 pg/mL in the first tertile, 54.9 ± 8.4 pg/mL in the second tertile, and 89.3 ± 17.8 pg/mL in the third tertile. Kaplan–Meier survival analysis indicated a significantly higher recurrence probability in patients with LPS levels in the third tertile. However, compared to the first tertiles, there were no significant differences in the second tertiles (Figure 1). The multivariable Cox regression analysis showed that Log-LPS, LAD, heart failure, and CITP were independent predictors of RAF (Table 2).

### 3.3. Association between Circulating LPS with Systemic Inflammation and Atrial Fibrosis

**Figure 2:**
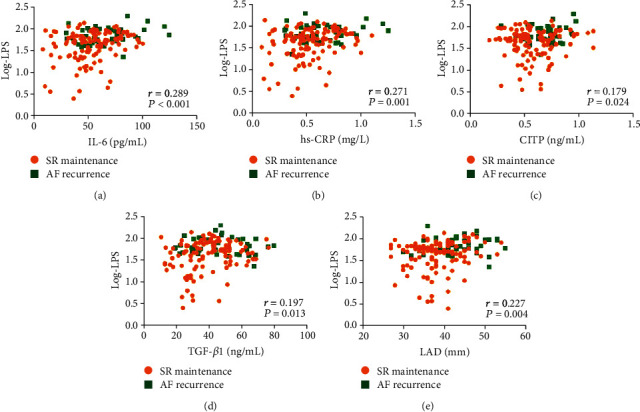
Scatter diagrams showing the association between the circulating LPS with the concentrations of inflammatory biomarker (IL-6 (a) and hs-CRP (b)), atrial fibrotic biomarkers (CITP (c) and TGF-*β*1 (d)), and LAD (e). Pearson's correlation coefficient and *P* values are indicated.

Log-LPS was significantly higher than that in patients with RAF (Table 1). Additionally, inflammatory biomarkers (hs-CRP and IL-6), atrial fibrotic biomarkers (CITP and TGF-*β*), and LAD were significantly increased in patients with RAF (*P* < 0.05, Table 1). Log-LPS was positively correlated with serum inflammatory biomarkers (IL-6: *r* = 0.289, *P* < 0.001; hs-CRP: *r* = 0.271, *P* = 0.001), fibrotic biomarkers (CITP: *r* = 0.179, *P* = 0.024; TGF-*β*1: *r* = 0.197, *P* = 0.013) and LAD (*r* = 0.227, *P* = 0.004) using Pearson's correlation analysis (Figures 2(a)–2(e)). Univariate linear regression analyses showed that Log-LPS was correlated with heart failure, LAD, hs-CRP, IL-6, CITP, and TGF-*β* (*P* < 0.05, Table 3). Subsequent multivariate analysis identified that heart failure, IL-6, and hs-CRP were independently related to Log-LPS (*P* < 0.05, Table 3).

## 4. Discussion

Accumulating evidence has shown that gut dysbiosis is related to the progression of RAF. The present study confirmed the findings that the gut microbiota-derived metabolite LPS is significantly positively correlated with RAF. Baseline LPS levels were higher in the RAF group than in the sinus rhythm group. In addition, we demonstrated that circulating LPS levels may promote RAF by increasing systemic inflammation and atrial fibrosis.

In this study, we found a RAF rate of 27.7% after ablation during the one-year follow-up, which is in accordance with other experimental data. This study is consistent with the meta-analysis conducted by Turagam et al., who found a rate of 27.5% RAF in 1212 AF patients after one-year follow-up [13]. Similar evidence has been reported in 256 AF patients to evaluate the effect of perindopril on RAF. In the subgroup of 128 patients treated with placebo, the recurrence rate of atrial fibrillation was 28.5% during the one-year follow-up [14]. However, another prespecified study from the CABANA Trial showed that 12.6% of ablation patients experienced symptomatic AF, and atrial tachycardia occurred in 36.4% of patients [15]. Overall, our results are consistent with the findings of these studies.

Considerable evidence has indicated that disordered gut microbiota contributes to RAF. Catheter ablation has been used as a first-line treatment strategy. However, the high recurrence rate postablation requires the identification of novel biomarkers to select optimal patients to improve clinical outcomes. The novel finding of the current study is that the patients with RAF disclosed higher baseline LPS during follow-up. In particular, we demonstrated that the circulating levels of patients in the third tertile of LPS (> 67.3 pg/mL) had the highest risk of RAF. Our results extend current knowledge on the effect of gut microbiota-derived metabolites in RAF, suggesting that gut microbiota may not only play a pathogenetic role in AF development but may also affect clinical outcomes after ablation.

Inflammation and oxidative stress are central mediators of AF, which exacerbate cardiac remodelling and facilitate AF initiation [16]. Menichelli et al. [17] confirmed that circulating LPS could contribute to impaired antioxidant status in the ATHERO-AF study. In this study, we further assessed the association between LPS and inflammatory cytokines. Inflammatory biomarkers (hs-CRP and IL-6) and fibrotic biomarkers (TGF*β*1 and CITP) were measured, which have proved to be an important mechanism leading to RAF [18, 19]. Previous studies have confirmed that a potential elevation in circulating hs-CRP and IL-6 has a higher risk of RAF [20]. Deftereos et al. found that colchicine can effectively prevent early AF recurrence by decreasing the levels of inflammatory mediators such as IL-6 and CRP [21]. Animal experiments found that LPS can stimulate M1 macrophage polarization to produce various proinflammatory cytokines. Additionally, TLR4 activation by LPS triggers consecutive MyD88 and TRIF-dependent signalling pathways, which synergistically promote the proinflammatory response [22]. In a canine model, inflammation induced by LPS could promote connexin 43 expression and cause heterogeneous atrial conduction, thereby increasing the risk of recurrence [23]. Consistent with previous studies, our data also indicated a significant association between circulating LPS and hs-CRP and IL-6. These studies showed that increasing chronic inflammation may be an important mechanism of LPS-induced recurrence of atrial fibrillation.

Clinical and animal evidence supports the viewpoint that atrial fibrosis is the hallmark of atrial structural remodelling and contributes to the occurrence and perpetuation of AF. The main pathological feature of atrial fibrosis was increased and disordered collagen deposition. CITP generated by the hydrolysis of type I collagen fiber, a serological marker of type I collagen degradation, has been demonstrated to be connected with the occurrence of AF [24, 25]. In addition, the progressive accumulation of the extracellular matrix produced by cardiac fibroblasts under profibrotic stimuli such as angiotensin II (Ang II) and TGF-*β*1 plays a pivotal role in promoting atrial fibrosis [26, 27]. Existing studies recognize the critical role played by TGF-*β*1 in inducing myocardial fibrosis [28]. In animal experiments, Zhang et al. demonstrated that gut dysbiosis induced AF partly through increased concentrations of circulating LPS and activated the atrial NLRP3 inflammasome, which promoted atrial fibrosis [10]. The present data demonstrated that circulating LPS levels were positively associated with the serum TGF-*β*1 and CITP levels. Subsequent multivariate analysis verified a significant independent association between LPS and the CITP levels. Another finding was that LAD is positively correlated with serum LPS levels. These results may further support the hypothesis that LPS induces RAF by promoting atrial fibrosis.

In addition, other mechanisms may contribute to the connection between LPS and RAF. Indeed, LPS may contribute to recurrence of AF through acceleration of heart failure and may induce myocardial infarction and left ventricular dysfunction, both of which could increase the recurrence risk of AF [29, 30]. Furthermore, there is an evidence that elevated circulating levels of LPS are connected with MACEs in AF patients. In the present study, our results demonstrate a significantly positive association between circulating LPS and heart failure and hypertension. Collectively, these studies confirm that promoting the occurrence of RAF-related risk factors may be an indirect mechanism leading to AF recurrence.

### 4.1. Study Limitations

The present study has several limitations. First, serum biomarkers of inflammation and atrial fibrosis are not heart specific. In addition, the findings were more convincing if measuring these biomarkers in the atrial tissue. Because of this potential limitation, we made strenuous efforts to exclude those patients with conditions related to inflammation and fibrosis. Second, the sample size was limited. Hence, more prospective studies are necessary to investigate the differences between paroxysmal AF patients and persistent AF patients in the future. Finally, we could not exclude other mechanisms by which LPS may contribute to RAF.

## 5. Conclusion

In conclusion, the results support the role of gut microbiota in the prognosis of AF postablation by demonstrating that baseline circulating LPS is associated with recurrence of AF during the one-year follow-up. Furthermore, baseline circulating LPS levels are associated with systemic inflammation and fibrotic biomarkers. However, further animal experiments are necessary to elucidate the potential mechanism of LPS-induced systemic inflammation and atrial fibrosis.

## Figures and Tables

**Table 1:** Characteristics of subjects with AF study According to RAF.

Variables	All	SR maintenance	AF recurrence	
	(*n* = 159)	(*n* = 115)	(*n* = 44)	*P*
Age (year)	61.9 ± 10.1	61.3 ± 10.2	63.3 ± 9.8	0.265
Male, *n* (%)	98 (61.6%)	72 (62.6%)	26 (59.1%)	0.638
Persistent AF, *n* (%)	64 (40.3%)	42 (36.8%)	22 (50.0%)	0.121
Hypertension, *n* (%)	85 (53.5%)	56 (49.1%)	29 (65.9%)	0.052
Diabetes, *n* (%)	21 (13.2%)	13 (11.3%)	8 (18.2%)	0.252
CHD, *n* (%)	35 (22.0%)	24 (20.9%)	11 (25.0%)	0.574
Heart failure, *n* (%)	34 (21.4%)	13 (11.3%)	21 (47.7%)	< 0.001
BMI (kg/m^2^)	25.1 ± 3.1	24.8 ± 2.9	25.8 ± 3.5	0.082
Glu (mmol/L)	5.04 (4.64-5.79)	4.99 (4.64-5.79)	5.20 (4.59-5.82)	0.599
LDL (mmol/L)	2.39 ± 0.81	2.4 ± 0.8	2.5 ± 0.8	0.432
HDL (mmol/L)	1.07 (0.93-1.36)	1.08 (0.94-1.36)	1.06 (0.85-1.36)	0.437
TC (mmol/L)	4.11 ± 0.96	4.10 ± 0.94	4.1 ± 1.0	0.916
TG (mmol/L)	1.44 (0.99,2.25)	1.38 (1.01-1.36)	1.33 (0.98-2.05)	0.605
Cr (*μ*mol/L)	71 (58-81)	69 (57-80)	72 (60-86)	0.127
LAD (mm)	39.6 ± 6.1	38.4 ± 5.5	42.7 ± 6.3	< 0.001
Hs-CRP (mg/L)	0.55 (0.41-0.72)	0.52 (0.40-0.68)	0.57 (0.47-0.79)	0.064
LPS (pg/mL)	56.5 ± 29.5	51.5 ± 29.2	69.5 ± 26.2	0.001
IL-6 (pg/mL)	56.8 ± 22.3	53.5 ± 20.8	64.8 ± 23.0	0.004
CITP (ng/mL)	0.62 ± 0.19	0.59 ± 0.19	0.69 ± 0.15	0.004
TGF-*β*1 (ng/mL)	41.1 ± 15.1	39.5 ± 14.4	45.2 ± 16.3	0.030

CHD: coronary heart disease; BMI: body mass index; LAD: left atrial diameter; IL-6: interleukin-6; CITP: collagen type-1 C-terminal telopeptide; TGF-*β*1: transforming growth factor beta1; LPS: lipopolysaccharide.

**Table 2:** Multivariable Cox regression to predict RAF.

Variables	Hazard ratio (95% CI)	*P*
Age (year)	0.990 (0.954-1.027)	0.598
Persistent AF, *n* (%)	1.018 (0.520-1.992)	0.959
Hypertension, *n* (%)	1.117 (0.562-2.221)	0.752
Diabetes, *n* (%)	1.505 (0.596-3.796)	0.387
CHD, *n* (%)	0.794 (0.331-1.905)	0.606
Heart failure, *n* (%)	2.029 (1.030-3.997)	0.041
BMI (kg/m^2^)	1.065 (0.972-1.167)	0.178
LAD (mm)	1.064 (1.009-1.123)	0.022
Hs-CRP (mg/L)	1.689 (0.456-6.253)	0.433
IL-6 (pg/mL)	1.009 (0.995-1.023)	0.226
CITP (ng/mL)	6.841 (1.168-40.069)	0.033
TGF-*β*1 (ng/mL)	1.021 (0.998-1.045)	0.079
Log-LPS (pg/mL)	5.686 (1.055-30.635)	0.043

CHD: coronary heart disease; BMI: body mass index; LAD: left atrial diameter; IL-6: interleukin-6; CITP: collagen type-1 C-terminal telopeptide; TGF-*β*1: transforming growth factor beta1; LPS: lipopolysaccharide.

**Table 3:** Univariate and multivariable linear regression analysis for the correlation of between Log-LPS with anthropometric and biochemical variables.

Variables	Univariate			Multivariable		
	Standardized *β*	95% CI	*P*	Standardized *β*	95% CI	*P*
Age (year)	0.089	-0.002-0.008	0.267			
Male, *n* (%)	-0.057	-0.143-0.067	0.472			
Persistent AF, *n* (%)	0.132	-0.016-0.191	0.098			
Hypertension, *n* (%)	0.125	-0.020-0.183	0.116			
Diabetes, *n* (%)	0.121	-0.034-0.266	0.128			
CHD, *n* (%)	0.078	-0.062-0.184	0.328			
Heart failure, *n* (%)	0.255	0.081-0.323	0.001	0.138	-0.010-0.228	0.071
BMI (kg/m^2^)	0.007	-0.016-0.017	0.926			
Glu (mmol/L)	-0.030	-0.029-0.021	0.743			
LDL (mmol/L)	0.087	-0.028-0.099	0.275			
TG (mmol/L)	0.078	-0.027-0.080	0.328			
Cr (*μ*mol/L)	0.016	-0.002-0.003	0.837			
LAD (mm)	0.227	0.004-0.021	0.004	0.144	0.001-0.016	0.057
Hs-CRP (mg/L)	0.271	0.175-0.619	0.001	0.215	0.105-0.525	0.003
IL-6 (pg/mL)	0.289	0.002-0.007	< 0.001	0.182	0.001-0.005	0.016
CITP (ng/mL)	0.179	0.042-0.589	0.024	0.123	-0.036-0.469	0.092
TGF-*β*1 (ng/mL)	0.197	0.001-0.008	0.013	0.144	0.001-0.006	0.050

CHD: coronary heart disease; BMI: body mass index; LAD: left atrial diameter; IL-6: interleukin-6; CITP: collagen type-1 C-terminal telopeptide; TGF-*β*1: transforming growth factor beta1; LPS: lipopolysaccharide.

## Data Availability

The datasets used and/or analyzed during this study are available from the corresponding author on reasonable request.
